# Supplementary material for: PTSD, Comorbidities, Gender, and Increased Risk of Cardiovascular Disease in a Large Military Cohort
Source: medRxiv. 2024 Apr 15:2024.04.13.24305769. Preprint. [Version 1] doi: 10.1101/2024.04.13.24305769 (PMC11065026; doi:10.1101/2024.04.13.24305769)
Supplement: Supplement 1 [file media-1.pdf]

SUPPLEMENT: eTables 1-7

Supplement eTable 1

List and Brief Descriptions of Administrative Data Systems in the Army STARRS Historical Administrative Data Study (HADS) Included in the Current Study

Database Acronym	Description
DMDC/CTS	DEFENSE MANPOWER DATA CENTER (DMDC) / CONTINGENCY TRACKING SYSTEM (CTS): Collection of activation, mobilization, and deployment data. Provides information to DoD decision makers and includes a CTS Deployment File used for tracking the location of deployed personnel.
DMDC/Master Personnel & DMDC/Transaction files	DEFENSE MANPOWER DATA CENTER (DMDC) / MASTER PERSONNEL & TRANSACTION FILES: The Active-duty Master File provides an inventory of all individuals on active-duty (excluding reservists on active-duty for training) at a point in time. It is a standardized and centralized database of present and past members of the active-duty force. Personal data elements include social security number, education level, home of record, date of birth, marital status, number of dependents, race, ethnic group, and name. Military data elements include Service, pay grade, Armed Forces Qualification Test percentile (enlisted only), source of commission (officers only), military primary duty and secondary occupation, Unit Identification Code, months of service, duty location, Estimated Termination of Service date, basic active service date, date of current rank, pay entry base date, foreign language ability, and major command code.
MDR	MILITARY HEALTH SYSTEM DATA REPOSITORY (MDR): This database contains information about medical, dental, pharmaceutical, and ancillary claims data for both in network and purchased care as well as both inpatient and outpatient treatment. Data are collected on both Army personnel and their beneficiaries.
TMDS	THEATER MEDICAL DATA STORE (TMDS): Used to track, analyze, view and manage Soldier medical treatment information recorded in the theater of operations. Features of TMDS: accessibility and visibility of servicemembers' deployed medical records, outpatient and inpatient treatment records created in theater facilities, treatment records from other applications, reports on movement of patients, patient status and injury/illnesses.
TRAC2ES	TRANSCOM REGULATING AND COMMAND & CONTROL EVACUATION SYSTEM (TRAC2ES): A tracking system for all medical transfers across the world for all DOD services.

Supplement eTable 2

International Classification of Diseases, Ninth Revision–Clinical Modification (ICD-9-CM) Codes Used to Identify Cardiovascular Disease

Cardiac Grouping	Severity[a]	Number of Soldiers[b]	Included Diagnoses	ICD-9-CM Codes
Hypertension	5	49,656	Malignant Hypertension	401.00
			Benign Hypertension	401.10
			Hypertension, Unspecified	401.90
			Hypertensive Heart Disease	402
			Essential Hypertension	410
Coronary Artery Disease	4	1,335	Angina Pectoris	413
			Coronary Artery Disease	414.00
			Coronary Atherosclerosis	414.40
			Cardiomyopathy	425
			Cardiovascular Disease, Unspecified	429.20
			Peripheral Vascular Disease	433.90
			Atherosclerosis	440.00
Stroke	3	516	Occlusion and Stenosis	
			of Basilar Artery with Cerebral Infarction	433.01
			of Carotid Artery with Cerebral Infarction	433.11
			of Vertebral Artery with Cerebral Infarction	433.21
			of Multiple/Bilateral Precerebral Arteries with Cerebral Infarction	433.31
			of Other Precerebral Artery with Cerebral Infarction	433.81
			of Unspecified Precerebral Artery with Cerebral Infarction	433.91
			Cerebral Thrombosis with Cerebral Infarction	434.01
			Cerebral Embolism with Cerebral Infarction	434.11
			Cerebral Artery Occlusion with Cerebral Infarction	434.91
Myocardial Infarction	2	87	Myocardial Infarction, Acute, Anterolateral	410.00
			Myocardial Infarction, Acute, Anterior	410.10
Congestive Heart Failure	1	489	Congestive Heart Failure	428.00

[a] From least (5) to most (1) severe.

[b] Number of soldiers with this diagnosis as their most severe

Supplement eTable 3

International Classification of Diseases, Ninth Revision–Clinical Modification (ICD-9-CM) Codes Used to Identify Risk Factors

Risk Factor Grouping	Included Diagnoses	ICD-9-CM Codes
PTSD	PTSD	309.81
Metabolic risk factors	Diabetes Mellitus, II Controlled	250.00
	Diabetes Mellitus, II Uncontrolled	250.02
	Metabolic Syndrome	277.70
	Overweight and Obesity	278.00
Behavioral risk factors	Alcohol Withdrawal Delirium	291.00
	Alcohol-Induced Persistent Amnestic Disorder	291.10
	Alcohol-Induced Persisting Dementia	291.20
	Alcohol-Induced Psych Disorder with Hallucinations	291.30
	Idiosyncratic Alcohol Intoxication	291.40
	Alcohol-Induced Psych Disorder with Delusions	291.50
	Alcoholic Psychosis NEC	291.80
	Alcohol Withdrawal	291.81
	Alcohol Induced Sleep Disorder	291.82
	Other Specified Alcohol-Induced Mental Disorder	291.89
	Unspecified Alcohol-Induced Mental Disorder	291.90
	Alcohol Intoxication – Unspecified	303.00
	Alcohol Intoxication – Continued	303.01
	Alcohol Intoxication – Episodic	303.02
	Alcohol Intoxication – In Remission	303.03
	Alcohol Dependence NEC/NOS	303.90
	Alcohol Dependence NEC/NOS – Continuous	303.91
	Alcohol Dependence NEC/NOS – Episodic	303.92
	Alcohol Dependence NEC/NOS – In Remission	303.93
	Alcohol Abuse	305.00
	Alcohol Abuse – Unspecified	305.00
	Alcohol Abuse – Continuous	305.01
	Alcohol Abuse – Episodic	305.02
	Alcohol Abuse – In Remission	305.03
	Tobacco Use Disorder	305.1 – 305.13

Depressive disorders	Major Depressive Disorder, Single Episode	296.20 – 296.29
	Major Depressive Disorder, Recurrent Episode	296.30 – 296.39
	Atypical Depressive Disorder	296.82
	Unspecified Episodic Mood Disorder	296.90
	Other Specified Episodic Mood Disorder	296.99
	Dysthymic Disorder	300.40
	Neurasthenia	300.50
	Adjustment Disorder with Depressed Mood	309.00
	Prolonged Depressive Reaction	309.10
	Depressive Disorder NOS	311.00
	Misery and Unhappiness Disorder	313.10
Sleep disorders	Nonorganic Sleep Disorder, NOS	307.40
	Transient Insomnia	307.41
	Persistent Insomnia	307.42
	Transient Hypersomnia	307.43
	Persistent Hypersomnia	307.44
	Circadian Rhythm Sleep Disorder, Nonorganic	307.45
	Sleep Arousal Disorder	307.46
	Sleep Stage Dysfunction, NOS	307.47
	Repetitive Intrusions of Sleep	307.48
	Nonorganic Sleep Disorder, Other	307.49
	Sleep Disorder/Disturbance	780.50
	Insomnia with Sleep Apnea	780.51
	Insomnia, Unspecified	780.52
	Hypersomnia with Sleep Apnea	780.53
	Hypersomnia, Unspecified	780.54
	Disruptions of 24-Hour Sleep-Wake Cycle	780.55
	Dysfunctions of Sleep Stages or Arousal	780.56
	Unspecified Sleep Apnea	780.57
	Sleep Related Movement Disorder, NOS	780.58
	Insomnia, Other	780.59

Note. NEC = Not Elsewhere Classifiable; NOS = Not Otherwise Specified

Supplement eTable 4

Overall Crude Rates of Hypertension Only and Arteriosclerotic Cardiovascular Disease per 1,000 Person-Years by Posttraumatic Stress Disorder Diagnosis, Gender, and Age Group

	Cases	Total	Crude Rate	Cases	Total	Crude Rate
PTSD						
No	47,455	29,429,948	19.35	2,320	30,377,118	0.92
Yes	2,201	442,238	59.72	107	508,460	2.53
Total	49,656	29,872,186	19.95	2,427	30,885,578	0.94
Gender						
Women	5,638	4,130,300	16.38	335	4,247,832	0.95
Men	44,018	25,741,886	20.52	2,092	26,637,746	0.94
Total	49,656	29,872,186	19.95	2,427	30,885,578	0.94
Age						
≤ 20 years	2,241	4,629,144	5.81	112	4,634,667	0.29
21-24 years	8,692	9,168,494	11.38	253	9,311,001	0.33
25-29 years	10,011	7,006,278	17.15	263	7,246,099	0.44
30-34 years	8,652	4,056,296	25.60	305	4,222,457	0.87
35-39 years	10,262	3,108,310	39.62	512	3,362,736	1.83
≥ 40 years	9,798	1,903,665	61.76	982	2,108,618	5.59
Total	46,656	29,872,186	19.95	2,427	30,885,578	0.94

Note. ASCVD = arteriosclerotic cardiovascular disease (coronary artery disease, stroke, myocardial infarction, congestive heart failure); PTSD = posttraumatic stress disorder

Supplement eTable 5

Crude Rates of Hypertension Only and Arteriosclerotic Cardiovascular Disease per 1,000 Person-Years by Posttraumatic Stress Disorder Diagnosis in Women and Men

		Hypertension Only			Arteriosclerotic CVD		
	PTSD	Cases	Total	Crude Rate	Cases	Total	Crude Rate
Women	No	5,475	4,066,208	16.16	321	4,179,748	0.92
	Yes	163	64,092	30.52	14	68,084	2.47
Men	No	41,980	25,363,740	19.86	1,999	26,197,370	0.92
	Yes	2,038	378,146	64.67	93	440,376	2.53
Total		49,656	29,872,186	19.95	2,427	30,885,578	0.94

Note. CVD = cardiovascular disease; PTSD = posttraumatic stress disorder

Supplement eTable 6

Crude Rates of Hypertension Only and Arteriosclerotic Cardiovascular Disease per 1,000 Person-Years by Posttraumatic Stress Disorder Diagnosis in Six Age Groups

		Hypertension Only			Arteriosclerotic CVD		
	PTSD	Cases	Total	Crude Rate	Cases	Total	Crude Rate
≤ 20 years	No	2,218	4,618,203	5.76	110	4,625,251	0.29
	Yes	23	10,940	25.23	2	9,416	2.55
21-24 years	No	8,325	9,059,783	11.03	244	9,203,093	0.32
	Yes	367	108,710	40.51	9	107,907	1.00
25-29 years	No	9,387	6,862,326	16.41	247	7,081,339	0.42
	Yes	624	143,951	52.02	16	164,760	1.17
30-34 years	No	8,205	3,975,320	24.77	292	4,117,442	0.85
	Yes	447	80,976	66.24	13	105,015	1.49
35-39 years	No	9,841	3,048,869	38.73	493	3,284,871	1.80
	Yes	421	59,442	84.99	19	77,865	2.93
≥ 40 years	No	9,479	1,865,446	60.98	934	2,065,121	5.43
	Yes	319	38,218	100.16	48	43,497	13.24
Total		49,656	29,872,186	19.95	2,427	30,885,578	0.94

Supplement eTable 7

Associations of First PTSD Diagnosis with Subsequent ASCVD Diagnosis Controlling for Hypertension in a Full Multivariable Model Among Regular Army Enlisted Soldiersl[a,b,c,d]

	OR	95% CI	χ^2, *p*
Gender			χ^2_1=1.4, *p*=0.2315
Female	0.9	[0.82, 1.05]	
Male	1.0	–	
Age (years)			χ^2_5=972.7, *p*<0.0001*
≤ 20	0.3	[0.24, 0.40]	
21–24	0.4	[0.32, 0.47]	
25–29	0.5	[0.42, 0.60]	
30–34	1.0	–	
35–39	2.0	[1.69, 2.27]	
≥ 40	5.1	[4.37, 5.86]	
Race			χ^2_4=1.5, *p*=0.8278
White Non-Hispanic	1.0	–	
Black	1.0	[0.94, 1.14]	
Hispanic	1.0	[0.84, 1.12]	
Asian	0.9	[0.75, 1.17]	
Other	1.1	[0.84, 1.36]	
Time in service (years)			χ^2_3=6.9, *p*=0.0754
1–2	0.8	[0.64, 1.08]	
3–4	1.0	[0.83, 1.20]	
5–10	1.0	–	
≥ 10	0.9	[0.74, 0.99]	
Rank			χ^2_4=15.1, *p*=0.0045
E1–E2	1.6	[1.26, 2.13]	
E3	1.2	[0.94, 1.56]	
E4	1.0	–	
E5–E6	1.0	[0.81, 1.14]	
E7–E9	0.9	[0.77, 1.15]	
Deployed[f]			χ^2_1=35.3, *p*<0.0001*
Yes	0.8	[0.70, 0.83]	
No	1.0	–	

MRF			χ^2_1=14.4, p=0.0001*
Yes	1.3	[1.13, 1.46]	
No	1.0	–	
BRF			χ^2_1=122.3, p<0.0001*
Yes	1.8	[1.59, 1.94]	
No	1.0	–	
Depression			χ^2_1=9.7, p=0.0018
Yes	1.2	[1.08, 1.41]	
No	1.0	–	
Sleep disorders			χ^2_1=21.6, p<0.0001*
Yes	1.4	[1.20, 1.55]	
No	1.0	–	
Hypertension			χ^2_1=592.2, p<0.0001*
Yes	3.3	[3.00, 3.63]	
No	1.0	–	
PTSD			χ^2_1=7.8, p=0.0052
Yes	1.4	[1.10, 1.68]	
No	1.0	–	

Note. ASCVD = arteriosclerotic CVD; CVD = cardiovascular disease; MRF = medical risk factors; BRF = behavioral risk factors; HADS = Army STARRS Historical Administrative Data Study; PTSD = posttraumatic stress disorder; STARRS = Army Study to Assess Risk and Resilience in Servicemembers.

*p < 0.00167 (sample size-adjusted alpha level).

[a] This sample of Regular Army enlisted soldiers were on active-duty at any point during 2004-2009 and is a subset of the total Army STARRS HADS.

[b] The sample included 2,427 ASCVD cases and 87,722 unweighted control person-months. All control person-months were assigned a weight of 362.1 to adjust for under-sampling.

[d] Multivariate model includes all of the variables listed plus historical time (using a dummy predictor variable for calendar month and year).
